# Genetic diversity and population structure of a global invader Mayweed chamomile (*Anthemis cotula*): management implications

**DOI:** 10.1093/aobpla/plab049

**Published:** 2021-08-27

**Authors:** Subodh Adhikari, Samuel R Revolinski, Sanford D Eigenbrode, Ian C Burke

**Affiliations:** 1 Department of Entomology, Plant Pathology and Nematology, University of Idaho, 875 Perimeter Drive MS 2329, Moscow, ID 83844, USA; 2 Department of Crop and Soil Sciences, Washington State University, Johnson Hall Rm. 115, PO Box 646420, Pullman, WA 99164, USA

**Keywords:** Gene flow, genotype, heterozygosity, inbreeding, invasiveness, migration, pairwise *F*_ST_, selfing

## Abstract

Mayweed chamomile (*Anthemis cotula*) is a globally invasive, troublesome annual weed but knowledge of its genetic diversity, population structure in invaded regions and invasion patterns remains unstudied. Therefore, germplasm from 19 *A. cotula* populations (sites) from three geographically distinct invaded regions: the Walla Walla Basin (located in southern Washington) and the Palouse (located in both northern Idaho and eastern Washington), Pacific Northwest, USA and Kashmir Valley, India were grown in the greenhouse for DNA extraction and sequencing. A total of 18 829 single-nucleotide polymorphisms were called and filtered for each of 89 samples. Pairwise *F*_ST_, Nei’s genetic distance, heterozygosity, Wright’s inbreeding coefficient (*F*) and self-fertilization rates were estimated for populations within and among the three regions with a total of 19 populations comprised of 89 individuals. Overall measurements of genetic variation were low but significant among regions, populations and individuals. Despite the weak genetic structure, two main genetic clusters were evident, one comprised of populations from Palouse and Kashmir Valley, the other comprised of populations from the Walla Walla Basin. Significant selfing was observed in populations from the Walla Walla Basin and Palouse but not from Kashmir Valley, indicating that Mayweed chamomile in the Pacific Northwest, USA could persist with low pollinator or pollen donor densities. Although *F*_ST_ values between the regions indicate Palouse populations are more closely related to Kashmir Valley than to Walla Walla Basin populations, based on Migrate-n analysis, panmixis was the most likely model, suggesting an unrestricted gene flow among all three regions. Our study indicated that Kashmir Valley populations either originated from or shared the origin with the Palouse populations, suggesting human-mediated migration of *A. cotula* between regions.

## Introduction

Due to global trade of plant materials and human movement, alien species have increasingly invaded new places, displaced native species, negatively affected biodiversity and altered ecosystem structure and function (Vilà *et al.* 2011; Van Kleunen *et al.* 2015; Kumar Rai and Singh 2020; Tallamy *et al.* 2020). Optimal management, prevention and mitigation of the impacts of invasive species on ecosystems depend upon understanding both the susceptibility of ecosystems to invasion and invasiveness of the invading species (Sterling *et al.* 2004; Vander Zanden *et al.* 2010). The invasiveness of alien weeds can be understood based on their genetic diversity and population structure (Richards *et al.* 2006), introduction history (Shaik *et al.* 2016; Hernández *et al.* 2019) and gene flow or migration (Li *et al.* 2019). Understanding of the genetics of weed populations may also provide insight into the long-term evolutionary consequences of agricultural management practices and potentially inform management decisions to improve sustainability.

Mayweed chamomile (*Anthemis cotula*) is an annual, bushy, ill-scented weed that originated in the Mediterranean region and eventually became a global invader (Kay 1971; Adhikari *et al.* 2020). Its global and local spread is believed to be anthropogenic, occurring via crop seed contamination and movement of farm equipment and other vehicles (Kay 1971; CABI 2018), but the exact history and pathways of worldwide migration are unknown. The weed is especially problematic in regions with Mediterranean-like climates such as the Pacific Northwest, USA and Kashmir Valley, India, although many other areas with similar climates have been infested or at risk of infestation by this weed (Shah *et al.* 2008; Lyon *et al.* 2017; Adhikari *et al.* 2020). *Anthemis cotula* prefers moist soil in arable lands, ditches, roadsides and other disturbed areas and is an economically important weed in agricultural lands (Adhikari *et al.* 2020). While it was not reported in India until about 50 years ago, it was introduced to the Pacific Northwest at least 144 years ago and currently is abundant across the region (Stewart *et al.* 1972; Global Biodiversity Information Facility 2020). Due to limited effective post-emergence herbicides in prevailing crops in the Pacific Northwest, managing *A. cotula* in broadleaf crops is difficult (Lyon *et al.* 2017; Adhikari *et al.* 2020). Anecdotal accounts from Pacific Northwest producers indicate that *A. cotula* pressure has been increasing and constitutes a barrier to diversification with cover crops and broadleaf rotational crops (e.g. pea, lentil, chickpea, canola) that producers are beginning to adopt in response to the changes in climate (Eigenbrode *et al.* 2013; O’Leary *et al.* 2018).

Despite these agronomic, economic and management issues, there has not been a systematic assessment of the genetic structure and diversity among *A. cotula* populations and their potential migration routes that presumably contribute to its continuing spread and invasiveness. It is unknown how *A. cotula* populations vary genetically and how this variation contributes to their invasiveness (Lee *et al.* 2008; Hufft and Zelikova 2016; Lucardi *et al.* 2020; Reatini and Vision 2020). Genetic variability in *A. cotula* could be narrow, due to the founder effect (Bakker *et al.* 2009; Neophytou *et al.* 2019), or it could be wide stemming from high diversity within or among single or multiple introductions (Smith *et al.* 2020) and ongoing diversification and adaptation.

The current abundance of *A. cotula* in the Pacific Northwest and reports of its developing recent herbicide resistance (Perez‐Jones *et al.* 2004; Intanon *et al.* 2011; Lyon *et al.* 2017; Heap 2020; S. Adhikari, I. C. Burke, S. D. Eigenbrode, unpubl. data) suggest it is adapting and becoming more difficult to manage. Although *A. cotula* is generally considered an obligate out-crosser, it may also be capable of self-fertilization (Kay 1971), which could increase its ability to colonize new habitats when founder populations are small (Rambuda and Johnson 2004; Kleunen *et al.* 2007; Bakker *et al.* 2009; Grant and Kalisz 2020). The status of selfing versus inbreeding in *A. cotula* populations, however, has not been assessed. Comparisons of genetic structure among populations within and among regions can shed light on its migration patterns during invasion and set a baseline for detecting subsequent evolution of the species and reintroduction events that could affect its management.

To address these gaps, we collected seeds from the 19 *A. cotula* populations from three key invaded regions: two in the Pacific Northwest, USA and one in Kashmir Valley, India and grew them in a common garden greenhouse setting for genetic analysis. We asked two questions: (i) What is the genetic diversity and population structure of these *A. cotula* populations? and (ii) Is there gene flow among *A. cotula* populations across regions? Based on its biology and ecology, possible repeated intrapopulation crossing events, and likely migration patterns, we expected genetic diversity to be low and population structure to be weak in *A. cotula* populations.

## Methods

### Seed collection and greenhouse common garden experiment

Seeds of 19 *A. cotula* populations (i.e. sampling sites or farms) were collected from three regions: the Palouse (located in both northern Idaho and eastern Washington; 13 populations) and the Walla Walla/Tucannon Basin (hereafter Walla Walla Basin, located in southern Washington; two populations) in the Pacific Northwest, USA; and Kashmir Valley, India (hereafter Kashmir Valley; four populations) (**see****Supporting Information—Table S1** for details of geographical locations for each of the 19 populations). The Walla Walla Basin and the Palouse regions are geographically separated **[see****Supporting Information—Table S1****]** and were sampled as separate regions because Walla Walla populations of *A. cotula* were collected from an area without chickpea production, whereas chickpeas are common in the Palouse (USADPLC 2016; USDA-AgMRC 2018). While the overall production practices in these two regions are similar, Walla Walla region in general has a warmer and drier climate **[see****Supporting Information—Table S1****]**, resulting in the earlier seasonal inputs.

On 27 February 2019, *A. cotula* seeds were planted in a greenhouse common garden using pots (13 cm × 13 cm × 13.5 cm) filled with a commercial greenhouse soil mix (75–80 % Canadian sphagnum peat moss, perlite and vermiculite; Premier Tech Horticulture Ltd, Alberta, Canada) under a 15-h photoperiod of sunlight and supplemental artificial light (photosynthetic photon flux = 595 µmol m^−2^ s^−1^) with an average temperature of 22.9 ± 0.26 (mean ± SE) °C and ambient humidity of 43.7 ± 9.5 % (mean ± SE). Twenty seeds from each of the 19 populations were planted into individual pots for a total of 95 pots, which were distributed on the greenhouse bench in a randomized complete block design. Pots were regularly watered as needed and not fertilized (additional details in Adhikari *et al.* 2021a, b).

### Sample collection for genotyping

Thirty days after seeding, when the seedlings were at the 3- to 5-leaf stage, leaf samples were collected from five randomly selected individuals of each population (*N* = 95) and stored in vials at −80 °C until further processing.

### DNA extraction and genotyping by sequencing

High-throughput automatic plant DNA extraction was performed with *A. cotula* samples. The frozen tissue samples were lyophilized for 48 h in a MultiDry benchtop freeze dryer (FTS Systems, Stone Ridge, NY, USA) and ground using a TissueLyser (Qiagen, Valencia, CA, USA). DNA was extracted from prepared tissue using a BioSprint 96 DNA Plant Kit (Qiagen) according to manufacturer’s instructions. Genotyping by sequencing libraries were prepared from *A. cotula* samples by ‘LGC Genomics’ following Elshire *et al.* (2011) using the MsII restriction enzyme. Barcode adapters were ligated to each sample and the samples were put into 48-plex library plates. Polymerase chain reaction was used to amplify samples on the plates. Libraries were sequenced in a single lane of the Illumina NextSeq 500 V2 (LGC Genomics) generating ~1.5 million 150-bp paired-end reads per sample.

After sequencing, the library groups were de-multiplexed with Illumina bcl2fastq software (Illumina 2019) allowing for up to two misreads on the barcodes. The groups were then de-multiplexed into samples according to the inline barcodes, where no mismatches were allowed. The adapter barcodes were then clipped and reads <20 bases in length were discarded. Reads with 5′ ends not matching the restriction enzyme were removed. Reads were quality trimmed from the 3′ end so that the average Phred quality score across 10 neighbouring bases was above 20. Finally, reads with missing base pairs or <20 bases in length were also discarded.

### Data analysis

#### Single-nucleotide polymorphism calling and filtering.

Contigs for single-nucleotide polymorphism (SNP) calling were created using Cluster Database at High Identity with Tolerance for expressed sequence tags (CD-HIT-est) (Li and Godzik 2006) to group all the reads from all the processed FASTQ files. A similarity threshold of 0.95 was used for running CD-HIT-est. After the duplicate sequences were removed, de-multiplexed filtered reads were aligned to the CD-HIT-est contigs using Burrows–Wheeler alignment (BWA-mem) (Li 2013) with default settings for paired-end reads. Sequence alignment map (SAM) files generated from the alignments were converted to binary alignment map (BAM) files and sorted using ‘SAMtools’ (Li *et al.* 2009). ‘FreeBayes’ (Garrison and Marth 2012) was used to call SNP from the BAM alignment files for the populations with the following settings differing from default settings; only calls for bi-allelic SNP, a minimum base quality score on reads of at least 10, minimum supporting allele ‘qsum’ of 10, read mismatch limit of 3, a minimum coverage of 5 and a minimum alternate count of 4. The variant call format (VCF) file generated from ‘FreeBayes’ was filtered so minor allele frequency (MAF) > 0.05, missing alleles < 70 % and quality score > 30 for each SNP using binary call format tools (‘Bcftools’) (Li 2011). Scripts in R statistical programming language were used to read the VCF file into an allelic dosage table and filter out markers with more than two alleles or completely heterozygote. Missing calls were imputed with a *k*-th nearest neighbour imputation using the ‘impute’ package (Hastie *et al.* 2020) from Bioconductor (Gentleman *et al.* 2004) in R. The SNP calling and filtering process resulted in 18 829 SNPs for each of the 89 samples collected. Six of 95 samples were removed from the analysis because either the samples were contaminated or based on the phenotypes (S. Adhikari *et al.*, unpubl. data; Adhikari *et al.* 2021a) they were determined to be scentless chamomile or false mayweed (*Tripleurospermum maritimum*).

To test for an association between geographic and genetic distance, and to perform analysis of molecular variance (AMOVA) with adaptive variants, the PCADAPT R package (Luu *et al.* 2017) was first used to obtain *P*-values for whether or not the variants could be considered adaptive (non-neutral). The qvalue package (Storey *et al.* 2021) from Bioconductor in R was used to correct for multiple testing and variants with a corrected *P*-value (i.e. *q*-value) below 0.1 were considered adaptive. Single-nucleotide polymorphism with a corrected *P*-value above 0.1 was considered neutral.

#### Genetic diversity and genetic/population structure analysis.

Analyses for genetic diversity and population structure were completed using the complete SNPs data set to provide a better picture of the overall variation and a more accurate depiction of population structure. Filtering by Hardy–Weinberg equilibrium can remove neutral variants that are related to population substructure (Chen *et al.* 2017); combining neutral and adaptive variants has been shown to provide more accurate population assignments (Batista *et al.* 2016); thus, for analyses unless otherwise specified the combined (adaptive and neutral) SNPs were used to assess population structure and genetic diversity. Pairwise *F*_ST_ and corresponding *P*-values were calculated between the populations of *A. cotula* using the ‘gl.fst.pop’ function of the DartR (Pembleton *et al.* 2013; Gruber *et al.* 2018) for 10 000 iterations. *P*-values were corrected for multiple comparisons (Bonferroni 1936). *F*_ST_, when calculated from many SNPs, remains unbiased when the sample sizes from each group are small or unequal if at low levels of differentiation (*F*_ST_ < 0.1) (Willing *et al.* 2012). Populations with sample sizes below four were not included in *F*_ST_ calculations in order to avoid upward bias (Willing *et al.* 2012). Analysis of molecular variance (Excoffier *et al.* 1992) was implemented using the ‘poppr’ (Dray and Dufour 2007; Kamvar *et al.* 2014) R package to breakdown the genetic variation into four components: between regions, between populations within region, within populations (i.e. between individuals or samples) and within individuals. Departure from panmixia and the components of variation were tested for significance using permutation implemented in the ‘randtest’ function of the ‘ade4’ R package (Dray and Dufour 2007). Analyses of molecular variance were performed on all populations (19) and on the regions (three: Kashmir, Palouse, Walla Walla Basin, in which those populations exist within), with subsets consisting of Kashmir/Walla Walla Basin and Kashmir/Palouse groupings tested as well. An additional AMOVA was performed within Palouse, to determine the variation explained by populations; it was done on only the Palouse because this region had 13 populations, compared to only two in Walla Walla Basin and four in Kashmir Valley. The AMOVAs were repeated using the adaptive SNPs.

Population groupings/admixtures were analysed using sparse non-negative matrix factorization (SNMF) (Hoyer 2004) and discriminant analysis of principal components (DAPC) (Jombart *et al.* 2010) implemented in ‘landscape and ecological association (LEA)’ (Frichot and François 2015) and ‘adgenet’ (Jombart 2008) R packages, respectively. The SNMF was used for getting a sense of the admixture while DAPC was used for hard clustering to clearly define groups. The ‘snmf’ function in ‘LEA’ was used to calculate the cross-entropy for each *K* number of clusters from 1 to 19. The *K* with the lowest cross-entropy was selected as the optimal number of clusters. The ancestry proportion matrix was calculated for *K* = 2 and *K* = 3. To use DAPC, we followed the protocol described by Jombart and Collins (2015) except testing for AIC (Akaike’s Information Criteria; a second metric), thus measuring both AIC and BIC (Bayesian Information Criteria) with *K* from 1 to 19.

A Linux command line tool ‘fineRADstructure’ (Malinsky *et al.* 2018) and unweighted pair group method with arithmetic mean (UPGMA) hierarchical clustering (Sokal and Michener 1958) of Nei’s genetic distance (Nei 1978) were used to determine the hierarchical structure of the populations. The ‘fineRAD structure’ package was used by running ‘RADpainter’ and ‘hapsFromVCF’ functions to convert the filtered VCF file into a haplotype file. The haplotype file was used to run ‘fineStructure’ with 100 000 burn-in iterations and 100 000 iterations after the burn-in sampling every thousand iterations. A Markov chain Monte Carlo (MCMC) tree was created using ‘fineStructure’ with 10 000 iterations. An additional tree was created by calculating Nei’s genetic distance in the ‘poppr’ R package (Kamvar *et al.* 2014) and then using UPGMA method on the Nei’s genetic distances to create the tree.

#### Genetic and geographical distance association.

Mantel test (Mantel 1967) and multiple regression of Moran’s Eigenvalue Maps (Wagner 2013) were used to determine spatial genetic associations of *A. cotula* in the Palouse region of the Pacific Northwest. For the spatial analysis, the Walla Walla Basin and Kashmir Valley regions, which had few populations, were removed leaving the 13 Palouse samples that were most of the *A. cotula* populations in our study. Pairwise *F*_ST_ values calculated from the neutral SNPs were used for the spatial analysis and the physical distance matrix for the Mantel test was calculated using the ‘pointDistance’ function (Hijmans 2020). We assume that fixed differences in allelic states between sites will be the product of genetic drift (Wright 1931, 1950). The ‘mantel’ function of the ‘vegan’ R package (Oksanen 2019) was used to calculate *P*-values with 100 000 permutations and the spearman correlation. The ‘memgene’ R package (Galpern *et al.* 2014) was used to perform multiple regression of Moran’s Eigenvalue Maps. The ‘mgQuick’ function of ‘memgene’ was used with coordinates as input, LonLat = TRUE, 10 000 forward permutations and 100 000 final permutations to detect spatial patterns that significantly explain genetic distances between sites.

#### Heterozygosity, Wright’s inbreeding coefficient and selfing rates.

Observed (*H*_O_) and expected heterozygosity (*H*_E_) were calculated using the ‘gl.basic.stats’ function in the ‘dartR’ library in R (Goudet 2005; Gruber *et al.* 2018). Wright’s inbreeding coefficient (*F*; Wright 1950) was calculated as: 1 − (*H*_O_/*H*_E_). Self-fertilization rates were estimated for each of 19 populations, three regions and for all regions combined using robust multilocus estimate of selfing (RMES) (David *et al.* 2007). Using a custom R script (Appendix 1), 1000 SNPs were selected at random and converted into RMES format and 10 000 iterations of RMES were used to generate *P*-values (Miller *et al.* 2014).

#### Migration patterns.

Migrate-n version 3.7.2 (Peter Beerli 1999; Beerli and Felsenstein 2001; Beerli and Palczewski 2010) was used to determine the optimal migration model and estimate the amount and direction of gene flow (i.e. migration rates) of *A. cotula* populations among Kashmir Valley, Walla Walla Basin and the Palouse regions. The ‘vcfR2migrate’ function of the ‘vcfR’ package (Knaus and Grünwald 2017) was used to convert the vcf file to the migrate file format, where bi-allelic markers with zero missing calls were kept, resulting in 129 SNPs. The four punitive models tested were: (i) migration between all populations or sites (Full model), (ii) migration between Walla Walla Basin and Palouse and from Walla Walla and Palouse to Kashmir Valley, (iii) migration from Pacific Northwest (Palouse and Walla Walla Basins combined into one population) to Kashmir Valley and (iv) a panmixis model where all the regions were treated as one panmictic population. The panmixis model was performed by coding all of the individuals as one population as specified by Beerli *et al.* (2019); thus, migration rates between sites were not estimated in this model. Migrate-n was run in the Debian Unix Windows 10 subsystem command line with 50 000 recorded genealogies sampled every 1000 steps and a burn-in of 10 000 genealogies. The temperatures (*T*) of the hot chains used to estimate likelihood approximations migrate were: *T* = 1, *T* = 1.5, *T* = 3, *T* = 1.0 × 10^6^. We estimated the potential migration pathways with the migration rate (*M*: migrant individuals/mutation rate) between regions. The estimated average effective genetic diversity (*θ* = 4*N*_e_*µ*; in diploid organisms, *N*_e_ is effective population size and *µ* is mutation rate) was calculated and the Bezier curve ln (likelihood) approximation from Migrate-n was used to select the most likely model, among our four models.

## Results

### Population assignment/clustering

**Figure 1.**
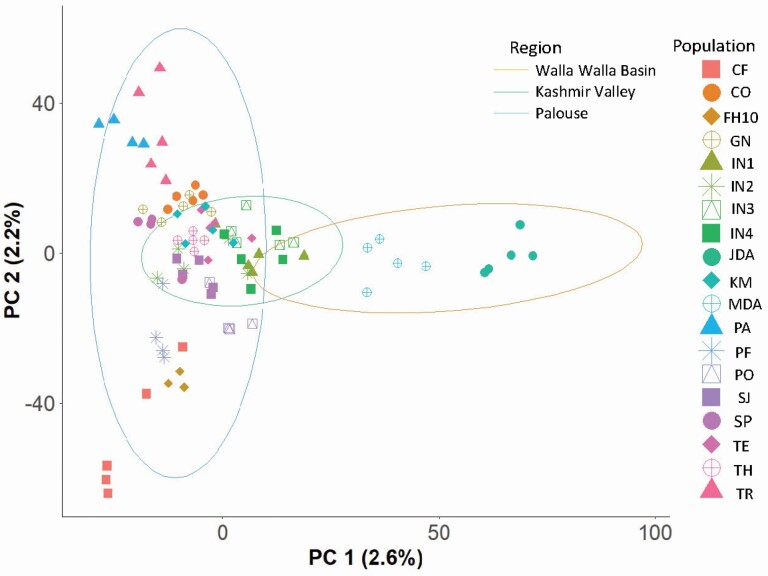
PCA plot of the clusters of 19 *A. cotula* populations (CF = R. J. Cook Agronomy Farm, CO = Colfax, FH10 = Foothill road, GN = Genesee, IN1 = Indian1, IN2 = Indian2, IN3 = Indian3, IN4 = Indian4, JDA = Dayton1, KM = Kambitsch, MDA = Dayton2, PA = Parker Farm, PF = Palouse Conservation Farm Station, PO = Potlatch, SJ = St. John, SP = Spillman Agronomy Farm, TE = Tensed, TH = Thornton, TR = Troy) and three regions (Walla Walla Basin = JDA and MDA; Kashmir Valley = IN1, IN2, IN3 and IN4; Palouse = CF, CO, FH10, GN, KM, PA, PF, PO, SJ, SP, TE, TH and TR). Centroids represent the 95 % confidence extent Gaussian distributions for regions.

**Figure 2.**
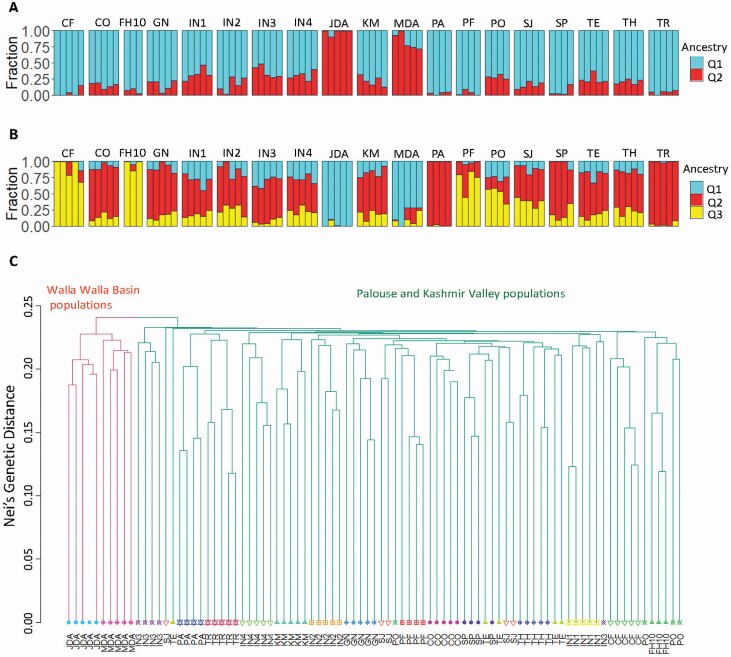
(A and B) Population structure of 19 *A. cotula* populations (*K* = 2 and 3). (C) UPGMA hierarchical tree of Nei’s genetic distance between samples. Leaves of the tree are colour/shape coded to the collection site and the branches are coloured by DAPC classification with two clusters (CF = R. J. Cook Agronomy Farm, CO = Colfax, FH10 = Foothill road, GN = Genesee, IN1 = Indian1, IN2 = Indian2, IN3 = Indian3, IN4 = Indian4, JDA = Dayton1, KM = Kambitsch, MDA = Dayton2, PA = Parker Farm, PF = Palouse Conservation Farm Station, PO = Potlatch, SJ = St. John, SP = Spillman Agronomy Farm, TE = Tensed, TH = Thornton, TR = Troy).

Discriminant analysis of principal components using AIC determined that the studied *A. cotula* populations had two clusters, while the BIC identified a single cluster to be optimal. Although LEA also identified one cluster to be optimal, with *K* = 2, its clustering matched the one found by DAPC–AIC (*K* = 2). The two clusters were samples from the Walla Walla Basin versus all other samples (Fig. 1). In both the PCA and LEA plots, Kashmir Valley populations clustered closer to Palouse populations than to Walla Walla Basin populations (Fig. 1). Results from the haplotyped genotype file using ‘fineRADstructure’ supported the clustering from DAPC–AIC because the first split on the maximum likelihood bootstrapped tree corresponded exactly to clusters of DAPC at *K* = 2 and *K* = 3. Most of the grouping in ‘fineRADstructure’ corresponded to sampling location (i.e. population). The grouping structure in the fineRADstructure plot (Fig. 2A and B), UPGMA tree (Fig. 2C) and co-ancestry structure plot **[see****Supporting Information—Fig. S1****]** were able to separate populations from Walla Walla Basin region from those of other two regions but further branching on the trees did not separate the populations from Palouse and Kashmir Valley.

### Selfing and genetic diversity

The estimated average selfing rate for the whole *A. cotula* population (i.e. all 19 samples combined) was 2.2 %. In region-wise estimation significant selfing was detected in the populations from the Walla Walla Basin and Palouse but not in Kashmir Valley populations based on the permutation test (Table 1). Estimated selfing rates were 2.5 % for the Walla Walla Basin, 2.1 % for the Palouse and 1.0 % for Kashmir Valley populations (Table 1). In population-wise estimation, selfing rates ranged from 0 % (five populations) to 13.8 % for the Parker Farm (Table 1).

**Table 1.** Observed (*H*_O_) and expected (*H*_E_) heterozygosity, inbreeding coefficient (*F*) and RMES selfing rates [s(g^2^)] across 19 *A. cotula* populations and three regions. CF = R. J. Cook Agronomy Farm, CO = Colfax, FH10 = Foothill road, GN = Genesee, IN1 = Indian1, IN2 = Indian2, IN3 = Indian3, IN4 = Indian4, JDA = Dayton1, KM = Kambitsch, MDA = Dayton2, PA = Parker Farm, PF = Palouse Conservation Farm Station, PO = Potlatch, SJ = St. John, SP = Spillman Agronomy Farm, TE = Tensed, TH = Thornton, TR = Troy.

	*H*_O_	*H*_*E*_	*F* (1 − *H*_*O*_/*H*_*E*_)	RMES	
				Selfing	*P*-value
Population (i.e. site)-wise					
CF	0.184	0.246	0.255	0.000	0.740
CO	0.161	0.251	0.358	0.054	0.002
FH10	0.184	0.218	0.158	0.067	0.005
GN	0.182	0.259	0.299	0.005	0.250
IN1	0.177	0.261	0.322	0.000	0.932
IN2	0.177	0.260	0.317	0.020	0.060
IN3	0.185	0.272	0.320	0.009	0.173
IN4	0.176	0.254	0.307	0.005	0.281
JDA	0.176	0.256	0.312	0.012	0.157
KM	0.179	0.259	0.308	0.000	0.692
MDA	0.191	0.271	0.297	0.081	0.000
PA	0.163	0.234	0.303	0.138	0.000
PF	0.181	0.238	0.240	0.005	0.264
PO	0.180	0.268	0.327	0.000	0.714
SJ	0.177	0.262	0.326	0.000	0.991
SP	0.179	0.247	0.273	0.003	0.350
TE	0.181	0.269	0.326	0.003	0.314
TH	0.180	0.262	0.312	0.000	0.477
TR	0.178	0.250	0.286	0.027	0.025
Region-wise					
Walla Walla Basin (*n* = 10)	0.184	0.271	0.322	0.025	0.007
Kashmir Valley (*n* = 20)	0.179	0.274	0.348	0.010	0.054
Palouse (*n* = 59)	0.178	0.273	0.349	0.021	0.000
Overall (*N* = 89)	0.180	0.273	0.340	0.018	NA

*H*_O_, *H*_E_ and *F* were similar among the three regions, and the overall *F*_IS_ across regions estimated at 0.3401 (Table 1). *H*_O_ ranged from 0.163 to 0.191, *H*_E_ ranged from 0.218 to 0.272 and *F* ranged from 0.158 to 0.358 among populations (Table 1).

### Population structure and genetic and geographical distance association

Overall measurements of genetic variation were low with the hierarchical AMOVA attributing 1.2 % of the genetic variation to sampling region, 7.0 % of the genetic variation to population within region and 27.8 % between samples within population (Table 2). Variation among regions, among populations (within region) and among individuals (within population) were all significant (*P* < 0.05) based on permutation testing with AMOVA (Table 2; **see****Supporting Information—Fig. S2**). A hierarchical AMOVA containing only Palouse and Kashmir Valley populations found that the region only explained 0.2 % of the genetic variation and that region was not a significant contributor to genetic variation (*P* = 0.15). Considering Palouse populations, 7.76 % of the genetic variation was explained by populations and population significantly contributed to overall genetic variation based on the AMOVA permutation test. The full hierarchical AMOVA performed with the adaptive SNPs yielded roughly similar results except that the percent explained (23 %) by region drastically increased **[see****Supporting Information—Table S2****]**.

**Table 2.** Analysis of molecular variance (AMOVA) table for between regions, within regions/between populations (sites), within populations/between samples and within samples comparisons. DF= degrees of freedom, SSD = sum of squared deviation, and MSD = mean squared deviation.

Hierarchical AMOVA (Full model)				Hierarchical AMOVA (Walla Walla Basin removed)				Hierarchical AMOVA (Walla Walla Basin and Kashmir Valley removed)			
	DF	SSD	MSD		DF	SSD	MSD		DF	SSD	MSD
Between regions	2	12 006	6003	Between regions	1	4961	4961				
Between pops.	16	72 831	4551	Between pops.	15	68 645	4576	Between pops.	12	55 282	4606
Between samples	70	206 580	2951	Between samples	62	182 245	2939	Between samples	46	133 505	2902
Within samples	89	140 516	1578	Within samples	79	124 280	1573	Within samples	59	92 660	1570
Total	177	431 933	2440	Total	157	380 132	2421	Total	117	281 448	2405
Variance components:											
	Sigma	Percent	*P*-value		Sigma	Percent	*P*-value		Sigma	Percent	*P*-value
Between regions	30.53	1.24	0	Between regions	4.78	0.20	0.15				
Between pops.	172.19	7.00	0	Between pops.	176.97	7.26	0	Between pops.	188.10	7.76	0
Between samples	686.16	27.81	0	Between samples	683.13	28.02	0	Between samples	665.89	27.47	0
Within samples	1578.83	63.98	0	Within samples	1573.17	64.53	0	Within samples	1570.52	64.78	0
Phi:											
Phi-samples-total	0.360			Phi-samples-total	0.355						
Phi-samples-pop	0.303			Phi-samples-pop	0.303			Phi-samples-total	0.352		
Phi-pop-region	0.071			Phi-pop-region	0.073			Phi-samples-pop	0.298		
Phi-region-total	0.012			Phi-region-total	0.002			Phi-pop-total	0.078		

**Figure 3.**
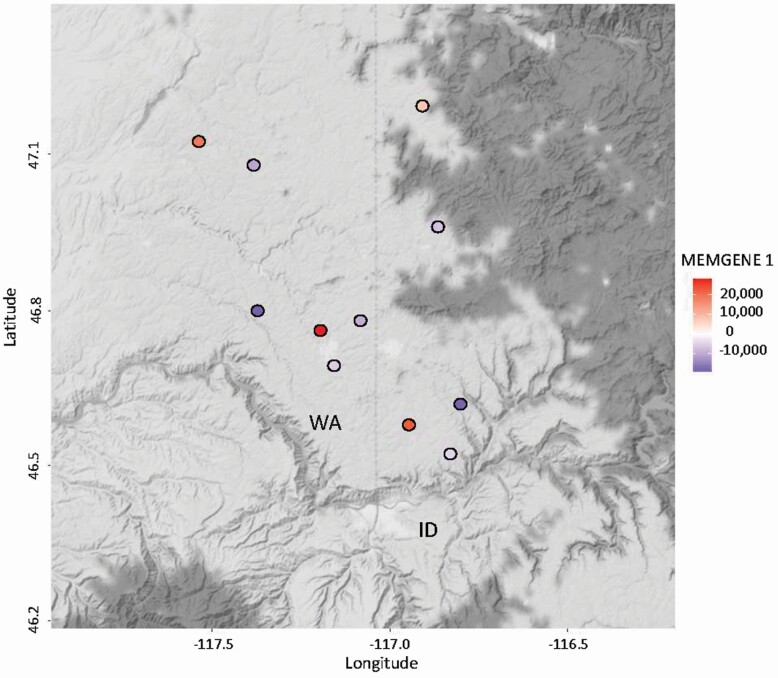
Positively (hot colours) and negatively (cool colours) correlated MEMGENE scores superimposed over geographical map of Pacific Northwest, USA (only Palouse populations are shown). MEMGENE-1 is the significant geo-genetic component.

Pairwise *F*_ST_ was calculated between populations and between regions to develop an understanding of the relative levels of genetic differences between samples from different populations or regions. The pairwise *F*_ST_ values between populations ranged from 0.018 between Tensed and Thornton populations to 0.147 between Dayton2 and Parker Farm populations, although all *F*_ST_ values were determined to be highly significant through bootstrapping (*P* < 0.0001; **see****Supporting Information—Table S3**). The pairwise *F*_ST_ values for the regions was 0.012 between the Palouse and Kashmir Valley, 0.045 between the Palouse and Walla Walla Basin and 0.041 between Walla Walla Basin and Kashmir Valley (*P* < 0.0001 based on bootstrapping) (Table 3). The overall *F*_ST_ between regions is estimated at 0.022 and between populations at 0.0762. Although significant differences were found between regions and between populations within the Palouse, a Mantel test detected no correlation between the genetic and physical distance between populations (*P* = 0.99; Fig. 3). The ‘memgene’ software, that was ran using coordinates and *F*_ST_ values, detected a spatial pattern (*P* < 0.05) where nearby sites were dissimilar to each other (Fig. 3), this is inconsistent with isolation-by-distance, as nearby sites were expected to be similar to each other if there was isolation-by-distance.

**Table 3.** Pairwise genetic distances or fixation index (*F*_ST_) among three regions for *A. cotula* populations. Upper triangle has *P*-values from 1000 bootstraps and the lower triangle has pairwise *F*_ST_ values from the dartR package.

	Kashmir Valley	Walla Walla Basin	Palouse
Kashmir Valley	NA	0	0
Walla Walla Basin	0.041	NA	0
Palouse	0.012	0.045	NA

### Migration and effective population size

**Figure 4.**
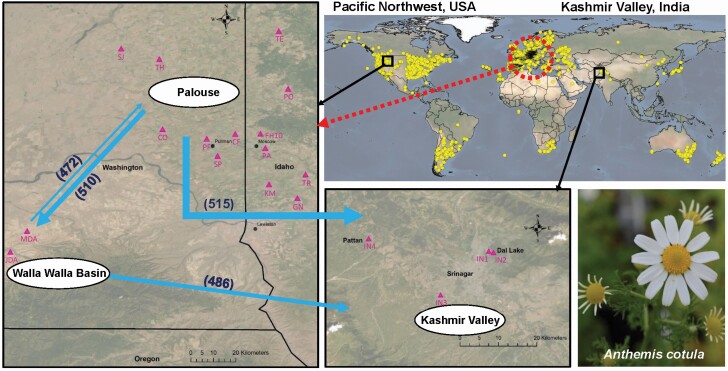
*Anthemis cotula* global distribution (yellow circles) map (Adhikari *et al.* 2020), study sites (pink triangles) and regions (white oval boxes), and possible migration routes (blue arrows) as estimated by Migrate-n. The arrow sizes are based on the estimated number of migrant individuals/mutation rate, given in the parenthesis. The dotted red circle around Mediterranean region indicates native range of *A. cotula* and the dotted red arrow indicates the possible introduction/migration to Pacific Northwest based on the literature (e.g. Mack and Erneberg 2002; CABI 2018). *Anthemis cotula* was introduced to North America likely as a contaminant in shipments of crop seed and forage (Adhikari *et al.* 2020) and was first reported in 1841 (GBIF 2020).

Using Bezier curve log-likelihood approximation from Migrate-n, panmixis was found to be the most likely model where all individuals are in one randomly mating population **[see****Supporting Information—Table S4****]**. The average effective genetic diversity (mutation- and ploidy-scaled effective population size) estimate (*θ*) for panmixis model was 0.076. Although the panmixis model was found to be the most likely, the model with three regions (i.e. Kashmir Valley, Palouse and Walla Walla Basin) with Walla Walla Basin and Palouse sourcing migration to Kashmir Valley (model 2) was used to generate estimates of genetic diversity for each population (i.e. region in this case). Using this approach, the path with the highest estimated migration rate (migrant individuals/mutation rate) was Palouse to Kashmir Valley and the lowest from Walla Walla Basin to the Palouse (Table 4; Fig. 4).

**Table 4.** Effective genetic diversity or theta (*θ*) estimates (on diagonals) and mutation-scaled migration (*M*) rates (off diagonals) between regions. No migration was assumed from Kashmir Valley, India to the Pacific Northwest (Walla Walla Basin and Palouse), USA.

Immigration\emigration among regions			
	Palouse	Kashmir Valley	Walla Walla Basin
Palouse	0.065	*	472
Kashmir Valley	515	0.049	486
Walla Walla Basin and Palouse	510	*	0.055
Theta for all populations (regions) combined (i.e. panmixis) = 0.076			

## Discussion

Genome-wide SNPs of 89 *A. cotula* individuals sampled from 19 populations that have invaded the Pacific Northwest, USA and Kashmir Valley, India revealed weak patterns of population genetic structure evidenced by significant *F*_ST_ values between and among populations and regions. *F*_ST_ values between populations and regions were mostly small and the bootstrapping found them to be significant. Although LEA and DAPC using BIC suggested a single cluster to be optimal, DAPC using AIC suggested two optimal clusters: one containing the Walla Walla Basin and the other with the remaining populations from Palouse and Kashmir Valley. This weak clustering indicates a weak population structure and that the populations have recently diverged, that ongoing migration promotes gene flow among the populations, or both (Loveless and Hamrick 1984; Edwards *et al.* 2020; Al Salameen *et al.* 2020).

Analysis of molecular variance (AMOVA) and pairwise *F*_ST_ revealed greater genetic variation associated with populations than with regions, consistent with a pattern of local adaptation of a generalist genotype, a process that can facilitate ecological invasions (Spitze and Sadler 1996; Gabriel *et al.* 2005). The higher percent of variation explained by region in the adaptive SNP AMOVA suggests that the differences observed between regions are due to differential selection on *A. cotula* populations between Walla Walla and the other two regions. Generalists from highly variable environments with high disturbance such as agricultural fields can evolve adaptive plasticity or genetic variation that promotes their establishment and persistence in local environments (Lee and Gelembiuk 2008). The low genetic differentiation in *A. cotula* populations among distant locations (Palouse, Pacific Northwest, USA and Kashmir Valley, India) suggests they are derived from one generalist population that can adapt locally to geographically and climatically diverse locales (Loveless and Hamrick 1984; Edwards *et al.* 2020). Accordingly, the distribution map indicates that *A. cotula* is already a globally invasive species and still expanding locally (Adhikari *et al.* 2020, 2021a,b).

In the Palouse region, despite the relatively high genetic variation among *A. cotula* populations (7.6 % of the total), there was no isolation-by-distance signal suggesting that differentiation through either selection, drift or a combination of both has occurred rapidly, overwhelming gene flow among populations. Rapid dissemination is likely at least facilitated by human transports. Compared to other globally invasive asters (e.g. *Centaurea solstitialis*: 0.15, estimated from SNPs; Eriksen *et al.* 2014 and *Conyza canadensis*: 0.93, estimated from microsatellites; Rosche *et al.* 2019), overall Wright’s inbreeding coefficient across *A. cotula* populations was moderate (0.34). Moderate inbreeding and significant selfing (2.5 and 2.1 %) in *A. cotula* populations from Palouse and Walla Walla Basins suggest there are prevalent local isolation and potential genetic bottlenecks. Additionally, relatively low rates of selfing in Kashmir Valley indicate that *A. cotula* populations are adaptively outcrossing with the capacity for self-fertilization when isolated or when pollinators are limited, potentially contributing to its invasiveness, as it does for other species (Stebbins 1957; Van Kleunen *et al.* 2008; Hartfield *et al.* 2017; Grant and Kalisz 2020). The low self-fertilization rate likely has increased differentiation between populations and individuals, leading to the overall inbreeding coefficients in populations. Selfing can improve the fitness of invading plants by reducing their dependence on pollinators in the early stages of invasion (Baker 1955, 1967; Pannell and Barrett 2017). Self-fertilization also increases genetic and phenotypic differences between populations (Willi *et al.* 2007).

Although Migrate-n analysis supports panmixis among the populations of *A. cotula* in this study, our model also suggests *A. cotula* emigration from Pacific Northwest, particularly more from the Palouse to Kashmir Valley, given that these populations are almost genetically indistinguishable. Circumstantial evidence is consistent with this invasion route. First, during late 1950s and 1960s, India was a major Pacific Northwest wheat importer; in 1967 only, USA exported 4.6 million metric tons (~$284 million) wheat to India (USDA Foreign Agricultural Service 2020), mostly via the Port of Portland terminal on the west coast of the USA (Donovan 2010). Both Walla Walla and Palouse in the Pacific Northwest export grain through the Port of Portland terminal to the world. Despite precautions, weed seeds, including *A. cotula*, regularly move between continents as contaminants in grain shipments (Shimono and Konuma 2008; Shimono *et al.* 2010, 2020; Conn 2012; Lehan *et al.* 2013; Early *et al.* 2016). Hence, *A. cotula* seeds from the Pacific Northwest could have been introduced to India as contaminants in grain shipments historically. Other commodities including pulses (particularly chickpeas), potentially contaminated with *A. cotula*, have been frequently shipped from the Pacific Northwest to India (USDA Foreign Agricultural Service 2020). Second, *A. cotula* was first reported in the Pacific Northwest 144 years ago but not in India until 50 years ago (Global Biodiversity Information Facility 2020; Stewart *et al.* 1972), which coincides with years of peak shipments of wheat between Pacific Northwest and India (USDA Foreign Agricultural Service 2020). Additionally, starting in 1980s, chickpeas are a frequent crop grown in the Pacific Northwest, particularly in the Palouse (USADPLC 2016; USDA-AgMRC 2018). Chickpea export to India began since 1990, which has been increasing significantly since then (USDA Foreign Agricultural Service 2020). One possible explanation of why Kashmir Valley populations are more closely related to Palouse populations than to Walla Walla Basin populations is that chickpea shipping could have been a vector allowing *A. cotula* to be transported from the Palouse to Kashmir Valley. Third, *A. cotula* is widely distributed in the Pacific Northwest but in India it is mostly confined to the Kashmir Valley (Adhikari *et al.* 2020) suggesting these regions are source and sink, respectively. However, it is unclear why other parts of India are less infested by *A. cotula* than the Kashmir Valley region. One possibility is that both Kashmir Valley and Pacific Northwest have similar (Mediterranean-like) climates, whereas other parts of India are mostly tropical. Other possibilities include multiple introductions or an admixed introduction from the USA, and our results closely mirrored the results of Von Boheemen *et al.* (2017) who explored the possibilities of multiple introductions from Europe or admixed introductions of *Ambrosia artemisiifolia*.

Despite revealing interesting genetic information among and within *A. cotula* populations, our study was limited by not having samples from the native range of *A. cotula*. While we have included *A. cotula* populations from its two key invading ranges (Pacific Northwest, USA and Kashmir Valley, India), we were unable to obtain seeds from the native range (Mediterranean region) which would have allowed us to compare the invasive genotypes with those of native ones. Previous studies have shown that invasive species that have both selfing and outcrossing strategies in their native ranges often have higher levels of self-pollination in the invasive range (Rodger and Johnson 2013). While we estimated selfing in invasive *A. cotula* populations, we lack this information in native populations. Also, although *A. cotula* is globally distributed (Adhikari *et al.* 2020), our study represented only two major continents: North America and Asia. Additionally, we were unable to collect historical details of crop seed trade records between the Mediterranean region (native range) and the USA, or between the Mediterranean region and India, that would have revealed the possible *A. cotula* global migration routes. Nevertheless, our study has provided an important baseline information on invasive *A. cotula* genotypes, and the future studies are required to investigate the historical details in *A. cotula* spreads via international crop seed trades and compare the phenotypic and genotypic traits of neophytes (i.e. introduced after the Columbian Exchange), archaeophytes (i.e. anciently introduced) and native populations.

### Management implications and future invasion potential

Since populations of *A. cotula* have some capacity for self-fertilization, they could invade and establish even with a limited number of propagules; thus, management efforts should aim to prevent invasion to new fields at local scale and to new regions at global scale. In a previous study, individuals (within a population) of *A. cotula* explained a higher proportion of variation than the populations for phenotypic traits (Adhikari *et al.* 2021b), which mirrored the genotypic variation in this study—that more effective management of crop seed movement and the discontinued use of field-scale selection pressure, like continuous use of a single herbicide, are needed.

At the global scale, *A. cotula* is historically known to be introduced or spread as an accidental seed contaminant (De Schweinitz 1832; Mack and Erneberg 2002; Lehan *et al.* 2013) and our data do not provide sufficient evidence to disprove the hypothesis of ongoing movement of the weed between Kashmir and the Pacific Northwest. Hence, stricter regulation of imported and exported crop seeds with weed risk assessment programmes (Lehan *et al.* 2013) is indicated again. High genetic variation within population and between population compared to low variation between distant regions suggests that invasive *A. cotula* populations could have originated from the same generalist genotypes that adapted to local conditions. If invasive *A. cotula* populations are originating from generalist genotype populations that can adapt to many locales, then many areas of the world can be at risk of invasion by *A. cotula*. Hence, measures such as improving *A. cotula* management at their source population sites and intervening contaminated shipments should be taken to prevent *A. cotula* from spreading via national and international (e.g. importing and exporting crops among countries) markets.

At the regional, farm or field scale, several human-mediated seed contamination pathways can occur during crop production, harvesting and crop handling (e.g. cleaning, grading, blending, storing) (Blanco-Moreno *et al.* 2004; Barroso *et al.* 2006; Wilson *et al.* 2016; Gao *et al.* 2018). Due to human-mediated movement of *A. cotula*, improved sanitary practices will be important for limiting the spread of herbicide resistance genes and other problematic genotypes. Established measures include limiting machinery movement, improving cleaning of equipment, ensuring use of certified seed and using new technology to clean grain of seeds during harvest (Gervilla *et al.* 2019; Norsworthy *et al.* 2020; Owen and Powles 2020). Trade and transportation of seed contamination of crops, especially with herbicide-resistant weeds, within and between continents (Shimono *et al.* 2010, 2020) can be particularly problematic. Future studies should focus on the origin and global migration routes of *A. cotula* to mitigate further spread.

## Supporting Information

The following additional information is available in the online version of this article—

**Table S1.** Geographical coordinates, elevation, and edaphic and climatic variables for locations where seeds of 19 *A. cotula* populations used for common garden experiment were collected.

**Table S2.** Analysis of molecular variance (AMOVA) table from adaptive single-nucleotide polymorphism (SNP) analysis between regions, within regions/between populations (sites), within populations/between samples and within samples comparisons.

**Table S3.** Pairwise genetic distances or fixation index (*F*_ST_) among *A. cotula* populations. *F*_ST_ values are below the diagonal, and *P*-values are above the diagonal.

**Table S4.** Migration models used in Migrate-n, log-likelihood Bezier curve values, log Bayes factor (LBF) and the model ranks.

**Figure S1.** Co-ancestry structure plot for 19 *A. cotula* populations.

**Figure S2.** Genotypic variations between and within *A. cotula* populations and among individual samples.

plab049_suppl_Supplementary_Materials

## Data Availability

All the raw data and R codes are publicly available in FigShare: https://doi.org/10.6084/m9.figshare.c.5510451.

## Notes

**Evolution & Diversity. Chief Editor:** Jeremy Beaulieu
