# Supplementary material for: Genetic diversity and population structure of a global invader Mayweed chamomile (Anthemis cotula): management implications
Source: AoB Plants. 2021 Aug 27;13(4):plab049. doi: 10.1093/aobpla/plab049 (PMC8403231; doi:10.1093/aobpla/plab049)
Supplement: plab049_suppl_Supplementary_Materials [file plab049_suppl_supplementary_materials.docx]

**Genetic diversity and population structure of a global invader Mayweed chamomile (*Anthemis cotula* L.): management implications**

Subodh Adhikari, Samuel R. Revolinski, Sanford D. Eigenbrode, Ian C. Burke

**Supporting Information**

**List of Supplementary Tables**

**Table S1:** Geographical coordinates, elevation, and edaphic and climatic variables for locations where seeds of 19 *A. cotula* populations used for common garden experiment were collected.

**Table S2:** AMOVA table from adaptive SNP analysis between regions, within regions/between populations (sites), within populations/between samples, and within samples comparisons.

**Table S3:** Pairwise genetic distances or fixation index (*F*_ST_) among A. cotula populations. *F*_ST_ -values are below the diagonal, and *P*-values are above the diagonal.

**Table S4:** Migration models used in Migrate-n, log likelihood Bezier curve values, log Bayes factor (LBF), and the model ranks.

**List of Supplementary Figures**

**Figure 1:** Co-ancestry structure plot for 19 *A. cotula* populations (CF = R. J. Cook Agronomy Farm, CO =Colfax, FH10 =Foothill road, GN =Genesee, IN1 = Indian1, IN2 = Indian2, IN3 = Indian3, IN4 = Indian4, JDA =Dayton1, KM = Kambitsch, MDA = Dayton2, PA =Parker Farm, PF = Palouse Conservation Farm Station, PO = Potlatch, SJ = St. John, SP = Spillman Agronomy Farm, TE = Tensed, TH = Thornton, TR = Troy) and three regions (Walla Walla Basin = JDA and MDA; Kashmir Valley = IN1, IN2, IN3, and IN4; Palouse = CF, CO, FH10, GN, KM, PA, PF, PO, SJ, SP, TE, TH, and TR.).

**Figure 2:** Genotypic variations between and within *A. cotula* populations and among individual samples.

**Supplementary tables and figures**

| **Table S1.** |  |  |  |  |  |  |  |  |  |
| --- | --- | --- | --- | --- | --- | --- | --- | --- | --- |
| Population | Latitude | Longitude | Elevation (m) | Precip. *  (mm) | Tmax. ^¥^  (^o^ C) | Tmin.^β^  (^o^ C) | Soil moisture^£^ (mm) | AET^€^  (mm) | CWD**  (mm) |
| **Palouse, Pacific Northwest, USA** | | | | | | | | | |
| Foothill road (FH10) | 46.7821 | -116.9804 | 819 | 699.89 | 14.49 | 2.16 | 127.16 | 44.15 | 29.29 |
| Genesee (GN) | 46.5233 | -116.8326 | 819 | 566.73 | 14.95 | 4.02 | 116.44 | 44.18 | 31.08 |
| Kambitsch Farm (KM) | 46.5788 | -116.9467 | 831 | 624.95 | 14.35 | 2.09 | 110.56 | 42.42 | 31.14 |
| Parker Farm (PA) | 46.7245 | -116.9610 | 811 | 681.81 | 14.91 | 2.42 | 123.64 | 44.05 | 30.30 |
| Palouse (PF) | 46.7615 | -117.1958 | 766 | 531.00 | 14.50 | 2.74 | 97.01 | 37.75 | 37.28 |
| Potlatch (PO) | 46.9620 | -116.8698 | 788 | 745.31 | 13.79 | 0.89 | 142.54 | 44.54 | 26.58 |
| Spillman Farm (SP) | 46.6930 | -117.1529 | 732 | 544.45 | 14.46 | 3.06 | 97.58 | 38.57 | 36.22 |
| St. John (SJ) | 47.1231 | -117.5389 | 630 | 456.52 | 15.48 | 2.37 | 47.11 | 32.90 | 52.73 |
| Tensed (TE) | 47.1928 | -116.9077 | 841 | 683.83 | 14.24 | 1.50 | 141.32 | 44.15 | 27.75 |
| Thornton (TH) | 47.0807 | -117.3858 | 701 | 491.06 | 14.93 | 2.33 | 79.83 | 34.44 | 42.18 |
| Troy (TR) | 46.6190 | -116.8004 | 753 | 616.58 | 15.13 | 2.79 | 122.96 | 44.08 | 30.17 |
| Colfax (CO) | 46.7997 | -117.3731 | 692 | 507.72 | 14.92 | 2.97 | 81.41 | 35.61 | 41.70 |
| Cook Farm (CF) | 46.7809 | -117.0817 | 788 | 624.95 | 14.31 | 2.40 | 114.85 | 41.55 | 31.96 |
| **Walla Walla Basin, Pacific Northwest, USA** | | | | | | | | | |
| Dayton.1 (MDA) | 46.3922 | -117.9158 | 616 | 478.97 | 16.69 | 4.23 | 44.00 | 31.64 | 53.53 |
| Dayton.2 (JDA) | 46.3086 | -117.9832 | 629 | 482.83 | 16.01 | 4.17 | 47.11 | 32.90 | 52.73 |
| **Kashmir Valley, India** | | | | | | | | | |
| Indian 1 (IN1) | 34.1289 | 74.8375 | 1591 | 766.50 | 20.53 | 8.96 | NA | NA | NA |
| Indian 2 (IN2) | 34.1256 | 74.8486 | 1583 | 766.50 | 20.53 | 8.96 | NA | NA | NA |
| Indian 3 (IN3) | 34.0264 | 74.7244 | 1601 | 777.90 | 20.13 | 8.86 | NA | NA | NA |
| Indian 4 (IN4) | 34.1581 | 74.5558 | 1597 | 737.30 | 20.11 | 9.06 | NA | NA | NA |
| Climate data are 30 years average (1981-2010).  Palouse: Palouse Conservation Field Station; Cook Farm: R.J. Cook Agronomy Farm; Spillman Farm: Spillman Agronomy Farm.  *Precip.: Mean annual Precipitation. ^¥^Tmax.: Mean annual maximum temperature. ^β^Tmin.: Mean annual minimum temperature. ^£^Soil moisture: Mean annual amount of water contained in the upper few meters of soil. ^€^AET: Mean annual actual evapotranspiration. **CWD: Mean annual water deficit between potential evapotranspiration and AET.  Source: https://climatetoolbox.org/tool | | | | | | | | | |

| **Table S2.** | | | | | | | | | | | |
| --- | --- | --- | --- | --- | --- | --- | --- | --- | --- | --- | --- |
|  | | | | | | | | | | | |
| Hierarchical AMOVA (Full model) | | | | Hierarchical AMOVA (Walla Walla Basin removed) | | | | Hierarchical AMOVA (Walla Walla Basin and Kashmir Valley removed) | | | |
|  | DF | SSD | MSD |  | DF | SSD | MSD |  | DF | SSD | MSD |
| Between regions | 2 | 572.64 | 286.32 | Between regions | 1 | 54.59 | 54.59 |  |  |  |  |
| Between pops. | 16 | 642.58 | 40.16 | Between pops. | 15 | 526.78 | 35.12 | Between pops. | 12 | 413.40 | 34.45 |
| Between samples | 70 | 1350.72 | 19.30 | Between samples | 62 | 1061.52 | 17.12 | Between samples | 46 | 756.72 | 16.45 |
| Within samples | 89 | 1129.00 | 12.69 | Within samples | 79 | 945.00 | 11.96 | Within samples | 59 | 631.50 | 10.70 |
| Total | 177 | 3694.93 | 20.88 | Total | 157 | 2587.89 | 16.48 | Total | 117 | 1801.62 | 15.40 |
| Variance components: | | | | | | | | | | | |
|  | Sigma | Percent | P-value |  | Sigma | Percent | P-value |  | Sigma | Percent | P-value |
| Between regions | 5.53 | 23.27 | 0.002 | Between regions | 0.31 | 1.83 | 0.127 |  |  |  |  |
| Between pops. | 2.24 | 9.44 | 0 | Between pops. | 1.95 | 11.80 | 0 | Between pops. | 1.99 | 12.76 | 0 |
| Between samples | 3.31 | 13.91 | 0 | Between samples | 2.58 | 17.74 | 0 | Between samples | 2.87 | 21.16 | 0 |
| Within samples | 12.69 | 53.38 | 0 | Within samples | 11.96 | 97.65 | 0 | Within samples | 10.70 | 100.00 | 0 |
| Phi: | | | | | | | | | | | |
| Phi-samples-total | 0.466 |  |  | Phi-samples-total | 0.288 |  |  |  |  |  |  |
| Phi-samples-pop | 0.207 |  |  | Phi-samples-pop | 0.177 |  |  | Phi-samples-total | 0.312 |  |  |
| Phi-pop-region | 0.123 |  |  | Phi-pop-region | 0.118 |  |  | Phi-samples-pop | 0.212 |  |  |
| Phi-region-total | 0.233 |  |  | Phi-region-total | 0.018 |  |  | Phi-pop-total | 0.128 |  |  |

| **Table S3.** | | | | | | | | | | | | | | | | | | |
| --- | --- | --- | --- | --- | --- | --- | --- | --- | --- | --- | --- | --- | --- | --- | --- | --- | --- | --- |
|  | IN1 | JDA | CF | CO | MDA | IN2 | IN3 | IN4 | GN | TE | TR | PO | SJ | KM | TH | SP | PF |  |
| IN1 |  | 0.000 | 0.000 | 0.000 | 0.000 | 0.000 | 0.000 | 0.000 | 0.000 | 0.000 | 0.000 | 0.000 | 0.000 | 0.000 | 0.000 | 0.000 | 0.000 |  |
| JDA | 0.086 |  | 0.000 | 0.000 | 0.000 | 0.000 | 0.000 | 0.000 | 0.000 | 0.000 | 0.000 | 0.000 | 0.000 | 0.000 | 0.000 | 0.000 | 0.000 |  |
| CF | 0.091 | 0.137 |  | 0.000 | 0.000 | 0.000 | 0.000 | 0.000 | 0.000 | 0.000 | 0.000 | 0.000 | 0.000 | 0.000 | 0.000 | 0.000 | 0.000 |  |
| CO | 0.054 | 0.093 | 0.088 |  | 0.000 | 0.000 | 0.000 | 0.000 | 0.000 | 0.000 | 0.000 | 0.000 | 0.000 | 0.000 | 0.000 | 0.000 | 0.000 |  |
| MDA | 0.058 | 0.047 | 0.097 | 0.065 |  | 0.000 | 0.000 | 0.000 | 0.000 | 0.000 | 0.000 | 0.000 | 0.000 | 0.000 | 0.000 | 0.000 | 0.000 |  |
| IN2 | 0.059 | 0.104 | 0.096 | 0.057 | 0.064 |  | 0.000 | 0.000 | 0.000 | 0.000 | 0.000 | 0.000 | 0.000 | 0.000 | 0.000 | 0.000 | 0.000 |  |
| IN3 | 0.044 | 0.074 | 0.084 | 0.049 | 0.049 | 0.051 |  | 0.000 | 0.000 | 0.000 | 0.000 | 0.000 | 0.000 | 0.000 | 0.000 | 0.000 | 0.000 |  |
| IN4 | 0.071 | 0.101 | 0.102 | 0.067 | 0.075 | 0.066 | 0.055 |  | 0.000 | 0.000 | 0.000 | 0.000 | 0.000 | 0.000 | 0.000 | 0.000 | 0.000 |  |
| GN | 0.060 | 0.104 | 0.094 | 0.054 | 0.068 | 0.059 | 0.053 | 0.068 |  | 0.000 | 0.000 | 0.000 | 0.000 | 0.000 | 0.000 | 0.000 | 0.000 |  |
| TE | 0.033 | 0.070 | 0.070 | 0.024 | 0.041 | 0.036 | 0.022 | 0.048 | 0.031 |  | 0.000 | 0.000 | 0.000 | 0.000 | 0.000 | 0.000 | 0.000 |  |
| TR | 0.084 | 0.121 | 0.113 | 0.066 | 0.089 | 0.087 | 0.069 | 0.096 | 0.074 | 0.057 |  | 0.000 | 0.000 | 0.000 | 0.000 | 0.000 | 0.000 |  |
| PO | 0.051 | 0.078 | 0.073 | 0.052 | 0.046 | 0.052 | 0.041 | 0.056 | 0.051 | 0.027 | 0.079 |  | 0.000 | 0.000 | 0.000 | 0.000 | 0.000 |  |
| SJ | 0.045 | 0.090 | 0.079 | 0.046 | 0.060 | 0.048 | 0.046 | 0.062 | 0.046 | 0.026 | 0.078 | 0.038 |  | 0.000 | 0.000 | 0.000 | 0.000 |  |
| KM | 0.062 | 0.099 | 0.096 | 0.058 | 0.073 | 0.063 | 0.056 | 0.070 | 0.057 | 0.042 | 0.080 | 0.056 | 0.055 |  | 0.000 | 0.000 | 0.000 |  |
| TH | 0.053 | 0.092 | 0.085 | 0.049 | 0.057 | 0.052 | 0.044 | 0.070 | 0.047 | 0.018 | 0.076 | 0.041 | 0.041 | 0.056 |  | 0.000 | 0.000 |  |
| SP | 0.069 | 0.125 | 0.102 | 0.065 | 0.086 | 0.071 | 0.057 | 0.092 | 0.068 | 0.038 | 0.094 | 0.072 | 0.059 | 0.079 | 0.061 |  | 0.000 |  |
| PF | 0.089 | 0.134 | 0.113 | 0.083 | 0.105 | 0.087 | 0.080 | 0.102 | 0.093 | 0.057 | 0.113 | 0.065 | 0.072 | 0.095 | 0.077 | 0.106 |  |  |
| Note: Populations (PA and FH.10) with less than 4 samples were removed from this analysis. Population names follow Table 1. | | | | | | | | | | | | | | | | | |  |

| **Table S4.** Migration models used in Migrate-n, log likelihood Bezier curve values, log Bayes factor (LBF), and the model ranks | | | |
| --- | --- | --- | --- |
| Models | Bezier  ln (likelihood) | LBF  (compared to full model) | Rank |
| Full Model (migration among all sites) | -2857 | 0 | 4 |
| No Migration from Kashmir Valley | -2805 | -53 | 3 |
| Pacific Northwest (as one population) to Kashmir Valley | -2703 | -155 | 2 |
| Panmixis | -2558 | -300 | 1 |


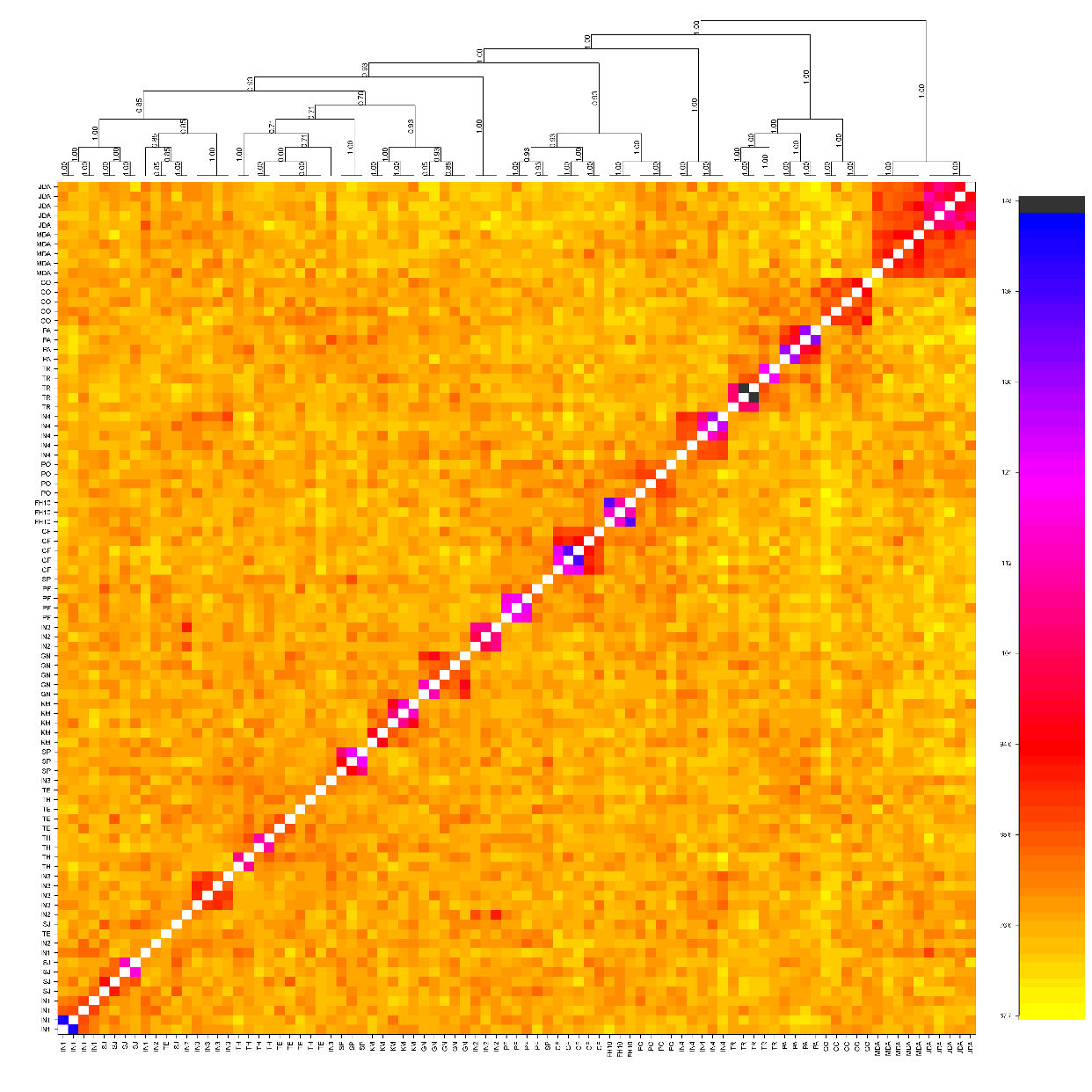


**Figure S1.**


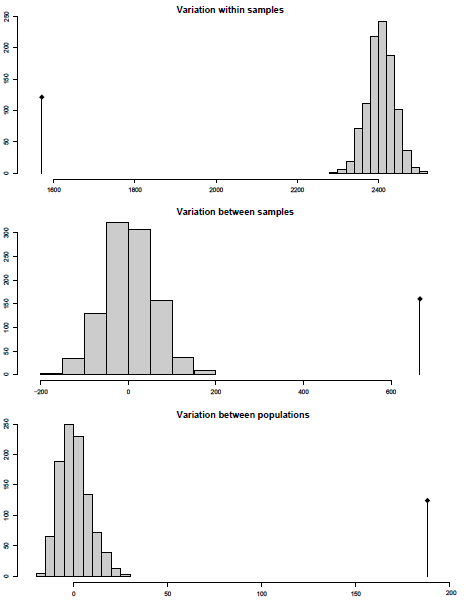


**Figure S2.**
